# Secondary Inflammation of the Parotid Gland

**Published:** 1887-04

**Authors:** 


Secondary Inflammation of the Parotid Gland.—As secondary inflammation of the parotid gland is thought generally to portend evil to the patient who has it develop subsequent to injuries and operation wounds, the following may prove of solace:

Mr. Stephen Padget reports sixty cases in the London *Lancet*, in all of which the primary lesion was in the abdomen or pelvis. Most of the patients recovered. In many there were no signs of septicæmia or pyæmia. The author draws the following conclusions:

1. That the parotid gland is related to the peritoneum.

2. That it is also related to the generative organs.

3. That an abdominal or pelvic lesion may be followed by parotitis without pyæmia.

4. That such a parotitis, if it occur later and with healthy kidneys, is usually followed by nursing.

Among these the parotitis followed—

1. The use of a catheter or sound in four instances.
2. Several cases followed labor, or induced abortion.
3. Several cases were from peritonitis, from injury or perforation.
4. Some followed operations, such as gastrotomy, operations on the cervix uteri, and especially ovariotomy. Following the latter they are not unusual.
5. Following pelvic celulitis and abscess. In one case reported, the woman had parotitis in six successive pregnancies, and one patient with amenorrhœa had a parotitis at each menstrual period.

